# Supplementary material for: Salivary Biomarkers and Oral Health in Liver Transplant Recipients, with an Emphasis on Diabetes
Source: Diagnostics (Basel). 2021 Apr 7;11(4):662. doi: 10.3390/diagnostics11040662 (PMC8067605; doi:10.3390/diagnostics11040662)
Supplement: Supplementary file 1 [file diagnostics-11-00662-s001.zip › Supplemetary material_LT_Diagnostics_final/Supplementary table S1_Diagnostics_LT.docx]

**Supplementary Table S1.** Salivary biomarkers in liver transplant recipients with and without diabetes after liver transplantation.

| **Biomarker** | **No diabetes** | **Diabetes** | **p** |
| --- | --- | --- | --- |
| No. of patients | 63 | 21 |  |
| MMP-8 [ng/ml] | 148.2 (125.0) | 194.6 (130.3) | 0.103 |
| TIMP-1 [ng/ml] | 251.2 (197.9) | 235.1 (172.0) | 0.487 |
| MMP-8/TIMP-1 molar ratio | 0.3 (0.3) | 0.5 (0.3) | 0.065 |
| Total protein [mg/ml] | 1.4 (0.4) | 1.6 (0.5) | 0.210 |
| Albumin [μg/ml] | 47.8 (50.9) | 63.1 (68.4) | 0.387 |
| IgA [μg/ml] | 40.0 (44.7) | 51.1 (40.2) | 0.068 |
| IgG [μg/ml] | 18.1 (18.9) | 20.7 (15.6) | 0.315 |
| IgM [μg/ml] | 5.4 (13.1) | 3.0 (2.5) | 0.941 |
| IL-1β [pg/ml] | 200.9 (169.5) | 278.5 (218.9) | 0.127 |
| TNF-α [pg/ml] | 7.6 (14.8) | 4.4 (6.4) | 0.584 |

Results are given as mean (SD), p-values correspond to Mann–Whitney U test.

Two patients were missing values in all biomarker groups.
