# Supplementary material for: Salivary Biomarkers and Oral Health in Liver Transplant Recipients, with an Emphasis on Diabetes
Source: Diagnostics (Basel). 2021 Apr 7;11(4):662. doi: 10.3390/diagnostics11040662 (PMC8067605; doi:10.3390/diagnostics11040662)
Supplement: Supplementary file 1 [file diagnostics-11-00662-s001.zip › Supplemetary material_LT_Diagnostics_final/Supplementary table S3_Diagnostics_LT.docx]

| **Supplementary Table S3**. Salivary biomarkers, salivary flow rate and periodontal inflammatory burden index scores compared with the use of immunosuppressive medication. | | | | | | | | | | |
| --- | --- | --- | --- | --- | --- | --- | --- | --- | --- | --- |
| **Biomarker/Parameter** | **CyA** | **Tacrolimus** | **p** | **Corticosteroid** | **No corticosteroid** | **p** | **MMF** | **AZA** | **No MMF or AZA** | **p** |
| No. of patients in group | 39 | 35 |  | 22 | 61 |  | 29 | 12 | 43 |  |
| MMP-8 [ng/ml] | 179.9 (172.4) | 93.1 (185.9) | 0.315 | 70.1 (120.8) | 164.4 (179.8) | **0.013** | 125.9 (190.6) | 203.2 (135.4) | 113.8 (181.8) | 0.143 |
| TIMP-1 [ng/ml] | 204.6 (177.5) | 148.8 (202.1) | 0.620 | 168.2 (106.8) | 206.5 (199.7) | 0.224 | 164.5 (233.4) | 275.0 (148.4) | 200.9 (152.0) | 0.437 |
| MMP-8/TIMP-1 molar ratio | 0.3 (0.3) | 0.2 (0.3) | 0.215 | 0.2 (0.3) | 0.3 (0.4) | 0.138 | 0.3 (0.6) | 0.4 (0.3) | 0.2 (0.3) | 0.265 |
| Total protein [mg/m] | 1.3 (0.7) | 1.6 (0.5) | **0.003** | 1.6 (0.8) | 1.4 (0.7) | 0.839 | 1.4 (0.6) | 1.6 (0.6) | 1.4 (0.8) | 0.484 |
| Albumin [μg/ml] | 37.1 (56.6) | 27.1 (37.0) | 0.988 | 33.3 (65.5) | 29.3 (45.1) | 0.316 | 29.5 (63.4) | 51.2 (55.9) | 25.8 (33.6) | 0.191 |
| IgA [μg/ml] | 28.0 (23.3) | 31.3 (33.5) | 0.226 | 33.4 (34.4) | 28.0 (26.4) | 0.341 | 25.9 (15.9) | 42.5 (55.3) | 31.4 (33.3) | 0.157 |
| IgG [μg/ml] | 11.9 (20.6) | 12.6 (22.8) | 0.490 | 11.1 (28.2) | 12.3 (19.2) | 0.233 | 11.6 (17.4) | 25.7 (31.3) | 10.9 (20.3) | 0.189 |
| IgM [μg/ml] | 2.3 (2.8) | 1.4 (3.7) | 0.753 | 2.5 (2.7) | 1.9 (3.4) | 0.243 | 1.4 (2.2) | 3.0 (2.5) | 2.4 (3.4) | 0.279 |
| IL-1β [pg/ml] | 290.7 (298.9) | 188.7 (255.8) | 0.685 | 130.8 (200.0) | 204.0 (259.2) | 0.174 | 196.4 (252.2) | 340.8 (298.6) | 101.7 (248.9) | **0.015^1^** |
| TNF-α [pg/ml] | 1.4 (9.5) | 1.7 (7.5) | 0.715 | 0.6 (1.5) | 3.0 (9.8) | **0.042** | 0.4 (3.7) | 10.1 (16.9) | 1.6 (3.9) | **<0.001^2^** |
| Unstimulated salivary flow rate [ml/min] | 0.3 (0.3) | 0.3 (0.4) | 0.379 | 0.4 (0.6) | 0.3 (0.4) | 0.705 | 0.5 (0.3) | 0.3 (0.4) | 0.3 (0.5) | 0.300 |
| Stimulated salivary flow rate [ml/min] | 1.6 (1.1) | 1.5 (2.0) | 0.818 | 1.7 (1.9) | 1.5 (1.3) | 0.872 | 2.0 (1.7) | 1.4 (1.2) | 1.5 (1.6) | 0.377 |
| PIBI score | 6.0 (12.0) | 3.0 (8.0) | 0.760 | 4.5 (11.3) | 3.0 (9.0) | 0.694 | 5.5 (9.5) | 8.5 (9.3) | 2.0 (7.8) | **0.045^3^** |
| Abbreviations: AZA = azathioprine; CyA = cyclosporin A; MMF = mycophenolate mofetil; PIBI = periodontal inflammatory burden index  Results are given as median (IQR), p-values correspond to Mann-Whitney U test or Kruskal Wallis test as appropriate  ^1^ Patients with AZA had significantly higher values of IL-1β than patients with MMF and patients with neither AZA or MMF.  ^2^ Patients with AZA had significantly higher values of TNF-α than patients with MMF and patients with neither AZA or MMF.  ^3^ Patients with AZA had significantly higher PIBI scores than patients with neither AZA or MMF. | | | | | | | | | | |
