# Supplementary material for: Salivary Biomarkers and Oral Health in Liver Transplant Recipients, with an Emphasis on Diabetes
Source: Diagnostics (Basel). 2021 Apr 7;11(4):662. doi: 10.3390/diagnostics11040662 (PMC8067605; doi:10.3390/diagnostics11040662)
Supplement: Supplementary file 1 [file diagnostics-11-00662-s001.zip › Supplemetary material_LT_Diagnostics_final/Supplementary Table S2_Diagnostics_LT.docx]

| **Supplementary Table S2.** Univariate and multiple linear regression analysis with the periodontal inflammatory burden index score as the dependent variable. | | | | |
| --- | --- | --- | --- | --- |
| **Independent variable** | **Univariate analysis** | | **Multivariate analysis** | |
|  | **B (95% CI)** | **p-value** | **B (95% CI)** | **p-value** |
| MMP-8 | 0.01 (-0.01 – 0.02) | 0.481 |  |  |
| TIMP-1 | -0.01 (-0.02 – 0.001) | 0.074 |  |  |
| MMP-8/TIMP-1 | 10.4 (4.6 – 16.2) | 0.001 | 11.7 (6.2 – 17.3) | <0.001 |
| Total protein | -2.6 (-6.7 – 1.4) | 0.200 |  |  |
| Albumin | 5.3x10^-5^ (-0.03 – 0.03) | 0.997 |  |  |
| IgA | -0.02 (-0.1 – 0.03) | 0.481 |  |  |
| IgG | 0.01 (-0.1 – 0.1) | 0.813 |  |  |
| IgM | 0.1 (-0.3 – 0.1) | 0.192 |  |  |
| IL-1beta | 0.01 (-0.01 – 0.02) | 0.364 |  |  |
| TNF-alfa | -0.02 (-0.2 – 0.1) | 0.733 |  |  |
| Resting salivary flow rate | 2.3 (-2.5 – 7.2) | 0.341 |  |  |
| Stimulated salivary flow rate | 2.1 (0.4 – 3.8) | 0.017 | 2.5 (0.9 – 4.0) | 0.002 |
| Smoking yes/no | 1.2 (-3.8 – 6.2) | 0.621 |  |  |
| Gender | -1.7 (-5.3 – 1.9) | 0.356 |  |  |
| Previous dental visits | -0.2 (-2.6 – 2.1) | 0.850 |  |  |
| Diabetes post LT yes/no | 3.3 (-0.8 – 7.3) | 0.111 |  |  |
| Own estimate of oral health | -0.4 (-3.2 – 2.3) | 0.750 |  |  |
| Level of education | 1.5 (-1.0 – 3.9) | 0.234 |  |  |
| Oral hygiene (cleaning between teeth yes/no) | 0.8 (-2.8 – 4.4) | 0.658 |  |  |
| No. of medications | -0.08 (-0.9 – 0.7) | 0.836 |  |  |
| Xerostomia | 1.1 (-2.5 – 4.7) | 0.538 |  |  |
| Abbreviations: LT = Liver transplantation | | | | |
